# Volumetric Imaging of Ex Vivo Oral Mucosa Specimens with Multi-Scale Wide Field-of-View Optical Coherence Tomography/Microscopy in Near-Infrared-II Window

**DOI:** 10.3390/diagnostics16111681

**Published:** 2026-05-29

**Authors:** Chuan-Bor Chueh, Shih-Jung Cheng, Hui-Hsin Ko, Ming-Che Tu, Ting-Hao Chen, Hsiang-Chieh Lee

**Affiliations:** 1 Graduate Institute of Photonics and Optoelectronics, National Taiwan University, Taipei 106, Taiwan; 2 Graduate Institute of Clinical Dentistry, School of Dentistry, National Taiwan University, Taipei 106, Taiwan; 3 Division of Oral and Maxillofacial Surgery, Department of Dentistry, National Taiwan University Hospital, Taipei 106, Taiwan; 4 Division of Oral and Maxillofacial Surgery, Department of Dentistry, National Taiwan University Hospital Hsin-Chu Branch, Hsinchu 302, Taiwan; 5 Department of Electrical Engineering, National Taiwan University, Taipei 106, Taiwan; 6 Molecular Imaging Center, National Taiwan University, Taipei 106, Taiwan

**Keywords:** optical coherence tomography, Near-Infrared-II, oral squamous cell carcinoma, intraoperative margin assessment, three-dimensional stitching, histological co-registration

## Abstract

**Background/Objectives:** Intraoperative margin assessments of oral squamous cell carcinoma (SCC) are fundamentally limited by sampling errors and freezing artifacts inherent to standard frozen section analysis. We developed a mobile, multi-scale, wide field-of-view (FOV) swept-source optical coherence tomography/microscopy (SS-OCT/OCM) system operating in the Near-Infrared-II (NIR-II) window (1.68 μm) to provide a rapid, non-destructive, volumetric evaluation of excised oral mucosal tissues. **Methods:** To correlate optical images with histopathology, we engineered a custom 3D-printed tissue cassette that physically mitigates macroscopic shrinkage during scanning and subsequent tissue fixation. A three-axis motorized assembly extends the effective imaging FOV without compromising resolution, while a custom 3D multi-resolution pyramid stitching algorithm synthesizes wide-FOV mosaics. **Results:** The customized cassette enabled precise, one-to-one spatial correlation between optical volumes and histopathology sections. Crucially, a 3 × 3 mosaic scan acquired with a 10× objective balanced imaging resolution and acquisition time, providing sufficient structural clarity to visualize basement membrane loss—a hallmark of SCC invasion. **Conclusions:** This 1.68 μm, fully automatic, multiscale SS-OCT/OCM platform demonstrates the feasibility of serving as a rapid, three-dimensional imaging tool for potential future use as an adjunct to conventional frozen sections.

## 1. Introduction

According to a 2024 report by the Taiwan Ministry of Health and Welfare, there were approximately 9000 new cases of oral cancer and 3500 deaths in 2022. Globally, the World Health Organization (WHO) estimates that there are approximately 400,000 new cases of oral cancer annually [1]. Alarmingly, the majority of patients are diagnosed at an advanced stage [2]. This delayed diagnosis is particularly concerning given the high malignant transformation rate of oral precancerous lesions. Consequently, there is a strong clinical need for techniques that enable the accurate, intraoperative identification of resection margins to ensure complete tumor removal.

Currently, surgeons primarily rely on clinical experience, visual inspection, and tactile palpation to identify oral inflammation, dysplasia, malignancies, and tumor boundaries. Standard practice typically involves resecting the lesion with a 5 mm margin beyond the visible boundary. While histopathological examination of biopsy specimens remains the diagnostic gold standard, standard formalin-fixed paraffin-embedded (FFPE) analysis requires multi-day processing, precluding its use for intraoperative surgical guidance. To achieve rapid feedback, surgeons often rely on frozen section analysis. However, this technique is fundamentally limited by freezing artifacts arising from ice crystal formation, which distort cellular morphology and compromise diagnostic accuracy. Furthermore, the high capital costs of equipment and the need for specialized on-site pathologists restrict the availability of frozen section analysis, particularly in smaller community healthcare facilities. Importantly, both standard histopathology and frozen section techniques are inherently susceptible to sampling errors. Because these methods evaluate only sparse, two-dimensional (2D) slices of a tissue section, there is a significant risk of false-negative margins, potentially leaving localized micro-tumors undetected in the unexamined region of the specimen.

While existing intraoperative imaging techniques, such as intraoperative computed tomography (iCT) [3], cone-beam CT (CBCT) [4,5], and C-arm fluoroscopy [6], are routinely employed during oral and maxillofacial surgeries, they are primarily optimized for bony structures rather than fine soft-tissue evaluation. Both iCT and CBCT present inherent risks of ionizing radiation and suffer from inadequate spatial resolution and soft-tissue contrast, making them insufficient for early detection and precise margin assessment of precancerous lesions [7]. Although C-arm fluoroscopy reduces radiation exposure, it further compromises diagnostic accuracy compared to iCT modalities. Consequently, an unmet clinical need remains for a radiation-free, high-resolution, and three-dimensional (3D) intraoperative imaging modality with the potential to aid in assessing tumor margins [8]. Ultimately, this system can lay the groundwork for future applications in evaluating potentially malignant disorders, such as confirming negative margins to minimize local tumor recurrence.

Optical coherence tomography (OCT), introduced by D. Huang et al. in 1991 [9], is a low-coherence interferometry technique that measures the intensity of backscattered light from tissues. This technique provides real-time, three-dimensional, high-resolution imaging with a typical penetration depth of 2–3 mm. OCT has been successfully adopted across various medical fields, including ophthalmology [10], cardiology [11], gastroenterology [12], and neurovascular imaging [13]. In the context of oral oncology, early investigations by P. Wilder-Smith et al. established the diagnostic potential of OCT for evaluating oral tissue [14]. Subsequent research has used custom OCT systems to differentiate normal from dysplastic mucosal tissues by identifying malignant architectural changes [15,16,17,18,19]. Various analytical approaches have been employed to characterize tissue scattering properties, such as measuring the light intensity gradient [20,21,22,23] or standard deviation (SD) [24,25,26], alongside automated criteria designed to enhance basement membrane visibility in cross-sectional OCT images [21,25]. While recent studies demonstrate a promising correlation between OCT findings and histopathology [27], a significant translational hurdle remains. Conventional histopathological preparation, including dehydration, embedding, sectioning, dewaxing, rehydration, and hematoxylin and eosin (H&E) staining, results in substantial tissue shrinkage and morphological distortion. This physical deformation fundamentally complicates the direct spatial co-registration of H&E-stained slides with the original, unadulterated OCT volumes [28,29]. Consequently, achieving a precise, one-to-one correlation of pathological features is exceedingly difficult, ultimately impeding specialists’ ability to establish reliable diagnostic criteria. Resolving this co-registration hurdle is a critical first step that is required to enable future studies to accurately interpret OCT images across varying degrees of oral oncology from early dysplastic changes (mild, moderate, and severe) to fully developed, invasive malignancies.

While optical coherence microscopy (OCM) systems successfully achieve a high resolution by combining coherence-gated OCT detection with high numerical aperture (NA) confocal spatial filtering [30,31], their application in oral oncology remains optically constrained. Most prior research relies on 1.3 μm OCT systems, which still suffer from signal attenuation when imaging through the thick, highly scattering keratinized layers of oral lesions. To overcome this fundamental limitation, our approach utilizes a swept-source laser operating within the Near-Infrared-II (NIR-II) window, a spectral regime proven to extend the ballistic region of light propagation through biological tissues [32,33,34,35]. Additionally, to address the persistent challenge of histological image registration, we designed a specialized tissue cassette to hold excised oral mucosal specimens. Featuring a stabilizing cover glass interface, this cassette enables uninterrupted, large-field-of-view (FOV) scanning while physically suppressing intrinsic tissue shrinkage induced by subsequent tissue fixation for H&E staining.

In this study, we present a novel, multi-scale, wide-FOV OCT/OCM system featuring a 1.68 μm swept-source laser. While long-wavelength OCT has shown broad potential, this specific system architecture has not been previously utilized for oral mucosa imaging. Engineered for clinical utility, the integrated mobile platform facilitates immediate ex vivo imaging of the oral mucosal specimens within the surgical suite. To process these wide-FOV volumetric datasets, we implemented a custom volumetric stitching and multi-resolution blending algorithm that seamlessly registers high-resolution sub-volumes while computationally eliminating hardware-induced artifacts. By utilizing this system to scan excised oral mucosal specimens housed in a custom-designed tissue cassette, this study explores the feasibility of the developed system for providing rapid, fully automatic, multi-scale, wide-FOV volumetric imaging of the entire excised oral mucosal tissue.

## 2. Materials and Methods

### 2.1. Multi-Scale SS-OCT/OCM System Architecture

**Figure 1:**
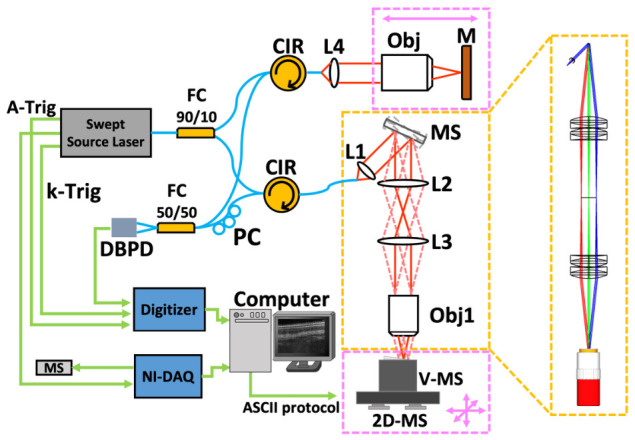
Schematic diagram of the multi-scale 1.68 μm SS-OCT/OCM system. FC: fiberoptic coupler; L1 and L4: collimating lenses; L2 and L3: lens relay pair; Obj1 and Obj: Objectives; M: mirror; MS: micro-electro-mechanical system (MEMS) scanning mirror; DBPD: dual-balanced photodetector; PC: polarization controller; CIR: fiberoptic circulator; V-MS: vertical motorized stage; 2D-MS: two-dimensional motorized stage. The light blue lines and red lines represent optical fiber connections and free-space optical beam paths, respectively. The green arrows denote electrical connections for triggers and control signals, while the magenta arrows indicate the directions of mechanical translation.

A schematic diagram of the custom-developed, multi-scale SS-OCT/OCM system is illustrated in Figure 1. The system is driven by a swept-source laser (HSL-1.7-90-B, Santec Corporation, Komaki, Japan) operating at a central wavelength of 1.68 μm, featuring an A-scan rate of 90 kHz and a wavelength-sweeping range of 175 nm. The laser output is directed into a 90/10 fiberoptic coupler, which splits the power between the sample and reference arms.

In the sample arm, approximately 90% of the optical power passes through a fiberoptic circulator and is collimated by a single aspheric lens (354220-C, Thorlabs, Inc., Newton, NJ, USA). The collimated beam is steered by a gimbal-less dual-axis micro-electro-mechanical system (MEMS) scanning mirror (Mirrorcle Technologies, Inc., Richmond, CA, USA). This configuration yields a highly compact profile with a fixed optical pivot point. To ensure the scanning beam is properly conditioned prior to entering the infinity-corrected objective, a custom telescope optical design was implemented. This optical relay accurately images the MEMS scanner’s pivot point onto the objective’s back focal aperture, achieving telecentric scanning and diffraction-limited performance across the tissue surface.

To enable multi-scale imaging, the sample arm incorporates three infinity-corrected objectives (5×, 10×, and 20× M Plan Apo NIR, Mitutoyo Corporation, Kawasaki, Japan) mounted on a motorized objective turret. This configuration enables seamless, real-time adjustments to lateral resolution, facilitating the exploration of NIR-II light–tissue interactions across varying structural scales. To execute the wide-FOV mosaicking protocol, the sample arm is equipped with a two-axis motorized stage (SR050B050B-T3A, Zaber Technologies, Inc., Vancouver, BC, Canada) integrated with a vertical motorized stage (VSR20A-T3A, Zaber Technologies, Inc., Vancouver, BC, Canada). This three-axis motorized assembly permits precise lateral translation and dynamic focal control, significantly expanding the effective FOV during high-magnification acquisitions.

In the reference arm, an identical collimator and a 5× objective (M Plan Apo NIR, Mitutoyo, Corporation, Kawasaki, Japan) are employed to mitigate the chromatic dispersion mismatches between the two interferometric arms. To account for the residual dispersion introduced when the sample arm utilizes a 10× or 20× objective, a numerical compensation method is applied during post-processing [36]. Furthermore, the reference arm features a single-axis motorized translation stage (MTS25-Z8, Thorlabs, Inc., Newton, NJ, USA) to automatically adjust the optical path length (OPL) to match that of each objective. Finally, the backscattered light from both arms is recombined via a 50/50 fiberoptic coupler. The interference signal is detected by a dual-balanced photodetector (BPD-200-HS, Santec, Corporation, Komaki, Japan) and digitized by a high-speed digitizer (ATS9350, AlazarTech, Inc., Pointe-Claire, QC, Canada) externally clocked by the swept-source laser.

### 2.2. Mobile Cart Integration and Clinical Software Engine

**Figure 2:**
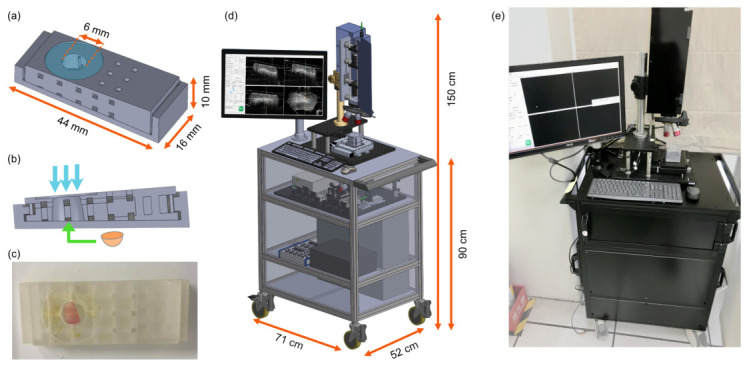
Hardware integration of the multi-scale OCT/OCM mobile cart and custom-developed tissue cassette. (**a**) 3D rendering of the custom-designed tissue cassette. (**b**) Cross-sectional schematic of the cassette illustrating the specimen placement, where the light blue arrows represent the incident light and the green arrow indicates the direction of tissue insertion. (**c**) Photography of the stereolithography (SLA) 3D-printed cassette housing with the excised oral mucosa specimen. (**d**) 3D rendering of the fully integrated mobile cart, designed for immediate ex vivo evaluation within the surgical suite. (**e**) Photography of the assembled, functional mobile cart system utilized for clinical imaging.

To translate the imaging system from a benchtop prototype to an intraoperative instrument, the entire optical, optomechanical, and computational framework—comprising imaging acquisition, real-time processing, and display modules—is integrated into a custom-designed mobile cart, as depicted in Figure 2d. With compact dimensions of 71 cm × 52 cm × 150 cm, the cart is highly maneuverable and suitable for navigation within space-constrained surgical suites and clinical examination rooms.

System control and data acquisition are governed by in-house software engine written in C++ that utilizes the Microsoft Foundation Classes (MFC) object-oriented library [37]. This software provides a unified graphical user interface (GUI) that seamlessly synchronizes the data acquisition card, the MEMS scanner, the motorized objective turret, the OPL-adjustment stage in the reference arm, and the three-axis motorized assembly in the sample arm. This integration is critical for executing the automated multi-scale, wide-FOV, mosaicking protocol while maintaining continuous focus within the imaged tissue specimens. Furthermore, to accommodate the immense data throughput generated by wide-FOV scanning and to ensure rapid intraoperative feedback for the surgeon, the signal processing and volumetric rendering pipelines are highly parallelized on a graphics processing unit (GPU) using the Compute Unified Device Architecture (CUDA) library.

### 2.3. Patient Cohort and Specimen Handling

Patients were recruited from the Division of Oral and Maxillofacial Surgery at National Taiwan University Hospital (NTUH) following protocols approved by the Institutional Review Board at the NTUH Research Ethics Committee (IRB No. 201711114DINA). The oral mucosa specimens were obtained via surgical resection under general anesthesia in the operating room or via incisional biopsy under local anesthesia in the outpatient clinic. This research is part of an ongoing clinical study with a target enrollment of approximately 100 patients. To demonstrate the technical capabilities of the developed platform, the representative imaging data presented in this feasibility study were drawn from an initial subset of 11 imaging specimens (6 normal mucosa and 5 SCC).

To preserve the tissue architecture during OCT/OCM imaging, specimens were housed in a disposable, custom-developed tissue cassette fabricated using stereolithography (SLA) 3D printing (Form 2, Formlabs, Inc., Somerville, MA, USA; Clear Resin), as depicted in Figure 2c. Upon excision, the specimens were gently rinsed to remove surface blood and excess fluids. This standardized preparation step is critical for minimizing unwanted surface pooling. Then, each specimen was placed directly into the cylindrical groove of the cassette. A cover glass (the blue transparent object in Figure 2a) was then secured over the cassette, providing physical contact to create a flattened imaging surface and mitigating tissue shrinkage during subsequent tissue fixation. To prevent specular reflection at the air–glass interface from coupling back into the collimator and saturating the photodetector, the cassette is engineered with the cover glass tilted 5° relative to the plane perpendicular to the optical axis. Furthermore, the cassette design incorporates lateral perfusion ports that allow for direct infiltration of 4% paraformaldehyde (PFA), thereby facilitating immediate tissue fixation without removal from the imaging holder.

### 2.4. Multi-Scale Mosaic Imaging Protocol and Histological Correlation

**Figure 3:**
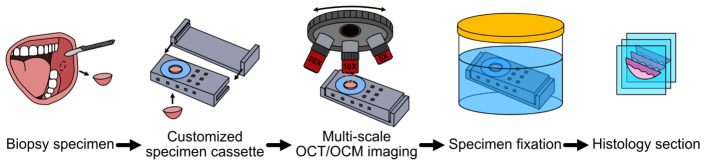
Schematic workflow of the ex vivo oral mucosal specimen processing, multi-scale imaging, and histological co-registration protocol.

The sequential experimental workflow integrating multi-scale mosaic imaging and histopathological co-registration is schematically illustrated in Figure 3. The imaging protocol began with a single, low-resolution volumetric scan using the 5× objective to localize the specimen within the cassette window macroscopically. Subsequently, the motorized objective turret was rotated to the 10× and 20× objectives to execute the high-resolution 3D mosaic scanning protocol. Concurrently, the motorized translation stage in the reference arm automatically adjusted the OPL to match the selected objective.

To image the entire resected tissue, the two-axis motorized stage translated the sample between each sub-volume acquisition, using 3 × 3 and 6 × 6 sub-volume grids for the 10× and 20× objectives, respectively. Both protocols utilized a 10% FOV overlap between adjacent sub-volumes to facilitate accurate subsequent image registration. Crucially, between each sub-volume acquisition, the vertical motorized stage dynamically adjusted the cassette’s elevation to compensate for the 5° tilt of the cover glass, ensuring that the imaged tissue remained in focus during mosaic scanning.

Immediately following image acquisition, the specimen was submerged in a 4% PFA solution. The cassette’s lateral perfusion ports allowed the fixative to displace water from the tissue rapidly. After a minimum of 16 h of in situ fixation, the specimen was removed and subjected to standard histopathological processing. The resulting H&E slides were subsequently digitized using an automated, high-resolution whole-slide scanner (SLIDEVIEW VS200, Olympus, Tokyo, Japan). Because the tissue was physically constrained during both imaging and fixation, the resulting H&E slides preserved a highly accurate, one-to-one spatial correlation with the OCT/OCM images. To enable direct visual correlation between these multi-scale OCT/OCM imaging datasets and the H&E sections, the acquired sub-volumes were subsequently processed using a custom wide-FOV 3D stitching pipeline, as described in the section below.

### 2.5. Volumetric Image Processing and Mosaicking

To generate a seamless, wide-FOV, volumetric representation of the imaged specimen, the acquired sub-volumes were processed with a custom-developed, automated 3D stitching algorithm. Let *m* and *n* denote the lateral spatial indices of an individual sub-volume within the overall mosaic grid, where *M* and *N* represent the total number of sub-volumes along each respective axis. As described previously, the total mosaic size (*M* × *N*) was 3 × 3 and 6 × 6 for the 10× and 20× objectives, respectively. To satisfy the Nyquist sampling criterion of the FOV while simultaneously adhering to the strict $2^{w}+1$ dimensional constraints (where *w* is a positive integer) required for symmetric 3D multi-resolution pyramid decomposition, the sub-volume tomograms $t^{m,n}$ were cropped and resized. Specifically, the 20× sub-volumes were resized to 513 × 257 × 257 voxels (~3.16 × 1.3 × 1.3 mm^3^), and the 10× sub-volumes were resized to 513 × 513 × 513 voxels (~3.16 × 2.5 × 2.5 mm^3^).

**Figure 4:**
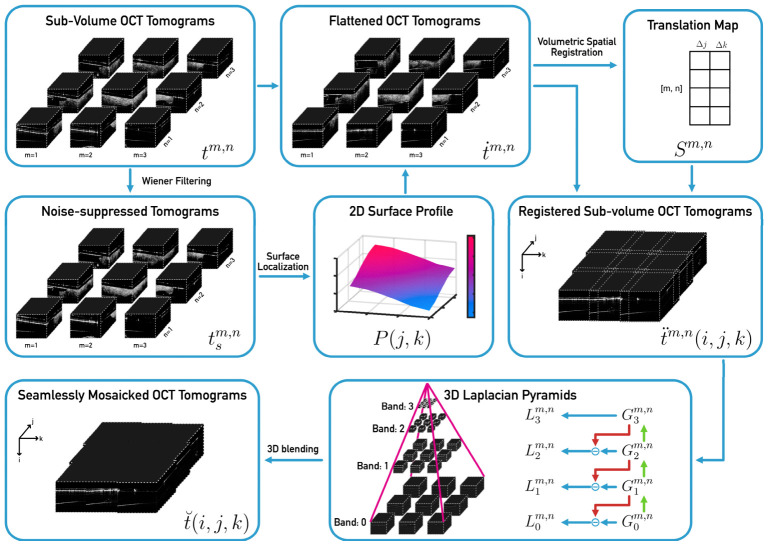
Schematic of the automated wide-FOV volumetric mosaicking pipeline. Raw sub-volume OCT intensity tomograms ($t^{m,n})$ first undergo Wiener filtering to generate noise-suppressed tomograms ($t_{s}^{m,n}$). Surface localization extracts a 2D surface profile (P(j,k)) which is used to flatten tomograms (${\dot{t}}^{m,n})$ and neutralize the hardware-induced tilt. Volumetric spatial registration yields a lateral translation map ($S^{m,n}$) to align the dataset, producing the precisely registered sub-volumes (${\ddot{t}}^{m,n}\left(i,j,k\right)$). To eliminate boundary seams and vignetting, these volumes undergo 3D multi-band blending; each is decomposed into a Gaussian (*G*) and Laplacian (*L*) multi-resolution pyramid. The band-pass Laplacian bands are spatially blended and collapsed to yield the final, seamlessly mosaicked volumetric reconstruction ($\overset{˘}{t}(i,j,k)$). Within the 3D Laplacian Pyramids schematic, the green arrows represent the *REDUCE* operation, red arrows indicate the *EXPAND operation*.

The complete processing pipeline for the wide-FOV mosaicking protocol is illustrated in Figure 4. Initially, an axial flattening protocol was applied to computationally compensate for surface tilting introduced by the 5°–tilted cover glass. This process involved applying a Wiener filter to generate speckle-suppressed tomograms $t_{s}^{m,n}$, followed by Canny edge detection to extract the air–glass interface precisely. A 2D surface boundary P(j,k) was calculated and utilized to axially align the A-lines, yielding an axially flattened tomogram ${\dot{t}}^{m,n}$ that successfully neutralized the hardware-induced tilt prior to lateral registration. Then, precise volumetric spatial registration was achieved via cross-correlation analysis on the overlapping regions between adjacent sub-volumes to determine the relative displacement, yielding a lateral translation map $S^{m,n}$. We denote the resulting surface-flattened and accurately registered sub-volume as ${\ddot{t}}^{m,n}\left(i,j,k\right)$, where *i*, *j*, and *k* represent the discrete 3D spatial coordinates (i.e., (z, x, y)).

During wide-FOV acquisition, the OCT signal intensity across individual sub-volumes is inherently non-uniform. This intensity discrepancy primarily arises from varying imaging depths due to a tilted cover glass within each volumetric scan, compounded by system-induced optical aberrations (e.g., vignetting) and the inherent sensitivity roll-off of the OCT system. Because brightness variations across sub-volumes are non-linear, conventional linear averaging techniques fail to seamlessly eliminate boundary artifacts, often leaving visible stitching seams. Therefore, 3D multi-resolution pyramid blending, adapted from the foundational multiresolution spline technique [38], was applied.

Let the original, full-scale OCT sub-volume be denoted as the base level (a=0) of the Gaussian pyramid:

(1) $$G_{0}^{m,n}\left(i,j,k\right)={\ddot{t}}^{m,n}\left(i,j,k\right)\;.$$

The subsequent low-pass filtered levels of the Gaussian pyramid are iteratively generated using a 3D REDUCE operator. For a given level a, utilizing a normalized 5 × 5 × 5 Gaussian generating kernel g, the reduction is computed as follows:

(2) $$\begin{array}{cc} G_{a}^{m,n}\left(i,j,k\right) & =REDUCE\left[G_{a-1}^{m,n}\right]\left(i,j,k\right)\\ & =\sum_{u=-2}^{2}\sum_{v=-2}^{2}\sum_{w=-2}^{2}g\left(u,v,w\right)G_{a-1}^{m,n}\left(2i+u,2j+v,2k+w\right). \end{array}$$

The same reduction operation is applied to generate the multiresolution spatial mask, $X_{a}^{m,n}\left(i,j,k\right)=REDUCE\left[X_{a-1}^{m,n}\right]\left(i,j,k\right).$ To isolate the specific spatial frequencies and structural details at each scale, a band-pass Laplacian pyramid $L_{a}^{m,n}$ is constructed by taking the difference between the Gaussian volume and the expanded version of the subsequent level a+1,

(3) $$\begin{array}{cc} L_{a}^{m,n}\left(i,j,k\right) & =G_{a}^{m,n}\left(i,j,k\right)-EXPAND\left[G_{a+1}^{m,n}\right]\left(i,j,k\right)\\ & =G_{a}^{m,n}\left(i,j,k\right)-G_{a+1,a}^{m,n}\left(i,j,k\right) \end{array}$$

(4) $$\begin{array}{cc} {G_{a,b}}^{m,n}\left(i,j,k\right) & =EXPAND\left[G_{a,b-1}^{m,n}\right]\left(i,j,k\right)\\ & =8\sum_{u=-2}^{2}\sum_{v=-2}^{2}\sum_{w=-2}^{2}g\left(u,v,w\right)G_{a,b-1}^{m,n}\left(\frac{i-u}{2},\frac{j-v}{2},\frac{k-w}{2}\right) \end{array}$$

with the top level A remaining equal to its Gaussian counterpart: $L_{A}^{m,n}\left(i,j,k\right)=G_{A}^{m,n}\left(i,j,k\right)$. Next, to seamlessly fuse the individual sub-volumes into a single wide-FOV mosaic, volumetric dataset, the overlapping Laplacian pyramids are blended at every spatial frequency level using the corresponding scaled spatial masks:

(5) $$LS_{a}\left(i,j,k\right)=\sum_{m=1}^{M}\sum_{n=1}^{N}X_{a}^{m,n}\left(i,j,k\right)L_{a}^{m,n}\left(i,j,k\right),$$

where $LS_{a}\left(i,j,k\right)$ represents the seamlessly blended Laplacian band at pyramid level a for the entire wide-FOV mosaic. The final, fully blended volumetric reconstruction, $\overset{˘}{t}\left(i,j,k\right)$, completely corrected for boundary seams and vignetting-induced variation in illumination intensities, is obtained by collapsing the blended pyramid through a recursive expansion and summation of all levels:

(6) $$\overset{˘}{t}\left(i,j,k\right)=\sum_{a=0}^{A}LS_{a,a}(i,j,k).$$

Here, the double subscript indicates that the seamlessly blended Laplacian at pyramid level a has been expanded a times to restore it to the base resolution prior to the summation operation.

## 3. Results

### 3.1. Characterization of the Multi-Scale SS-OCT/OCM System

**Figure 5:**
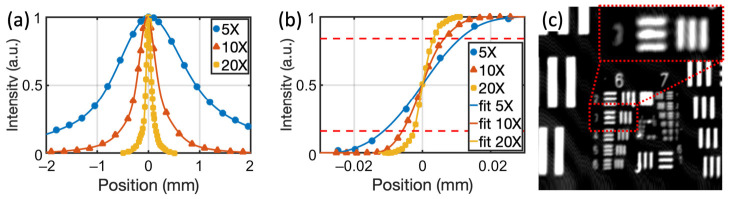
System optical characterization. (**a**) Confocal gate function for the 5× (blue), 10× (red), and 20× (orange) objectives. (**b**) Edge spread function for the corresponding objective lens obtained via the knife-edge resolution method. The solid line denotes the fitted curves, while the horizontal red dashed lines indicate the 16% and 84% maximum intensity thresholds used to determine the transverse spot size. (**c**) *En face* OCM image of the USAF 1951 resolution target acquired with the 20× objective lens. The red dashed boxes highlight a magnified view of Group 6, Element 3.

By placing a mirror in the sample arm and measuring the back-reflected light power from the various objectives, we characterized the confocal gate function for each objective, as shown in Figure 5a in a normalized manner. The depth of focus (DOF) for the objectives is defined by the full-width at half-maximum (FWHM) of the confocal gate function. The measured DOF values for each objective are summarized in Table 1.

Additionally, we imaged the U.S. Air Force (USAF) 1951 resolution target with three different objectives used in our multi-scale OCT/OCM system. By measuring the intensity profile of the resolution target’s square patterns, we derived the edge spread function for each objective (Figure 5b). The red dashed lines in Figure 5b indicate the 84% and 16% maximum intensity thresholds, respectively. By measuring the distance between these thresholds, which corresponds to the Gaussian spot $1/e^{2}$ radius, we calculated the transverse spot size FWHM (Table 1).

Furthermore, we present an *en face* OCM image of the USAF 1951 resolution target acquired using the 20× objective (Figure 5c). As highlighted by the red dashed box, the bars of Group 6, Element 3 are clearly resolvable, achieving a modulation transfer function of at least 32%. Finally, we determined the FOV for different objectives by imaging a multi-frequency grid distortion target (R1L3S3P, Thorlabs, Inc.) and counting the visible grids in the *en face* OCM image (Table 1).

### 3.2. Multi-Scale OCT/OCM Imaging of the Ex Vivo Oral Mucosal Specimens

To evaluate the feasibility of the developed multi-scale OCT/OCM imaging system, we acquired ex vivo images of human oral mucosal tissue, encompassing both squamous cell carcinoma (SCC) and adjacent normal regions. The system’s automated mosaic scanning protocol enabled efficient large-area evaluation, acquiring comprehensive multi-resolution imaging datasets (using 5×, 10×, and 20× objectives) within approximately 20 min per specimen. Following the acquisition, all volumetric reconstructions, 3D multi-resolution pyramid blending, and image processing were performed in MATLAB R2025a (MathWorks, Natick, Massachusetts) on a workstation with an Intel Core i7-9700K processor (3.6 GHz) and 64 GB of memory. Under this hardware configuration, the multi-resolution stitching algorithm leverages MATLAB’s parallel computing capabilities to maximize computational efficiency. Consequently, the total processing time required to construct a complete, wide-FOV mosaic volume is remarkably rapid, taking approximately 30 s for both the 10× and 20× volumetric datasets.

**Figure 6:**
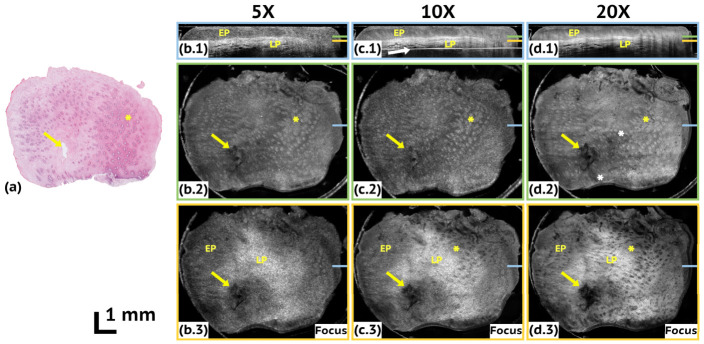
Multi-scale three-dimensional OCT/OCM images and corresponding H&E-stained histology of a healthy right mandibular gingiva specimen from a 53-year-old female. (**a**) Corresponding H&E-stained histology, which matches the *en face* image (**b.2**,**c.2**,**d.2**). (**b.1**,**c.1**,**d.1**) Cross-sectional OCT/OCM images acquired with the 5×, 10×, and 20× objectives, respectively. (**b.2**,**c.2**,**d.2**) *En face* OCT/OCM images extracted at a depth of 332 μm (indicated by the horizontal green lines in the cross-sections). (**b.3**,**c.3**,**d.3**) *En face* OCT/OCM images extracted at a depth of 570 μm (indicated by the horizontal orange lines). Note that across all magnification scales, the optical focal plane was positioned closer to this deeper plane (denoted by “Focus”). EP: epithelium; LP: lamina propria. Yellow asterisks (*): rete pegs. Yellow arrows: accidental needle puncture track. White asterisks (*): stitching artifacts. White arrow: specular reflections. Scale bars: 1 mm. The light-blue tick on the *en face* images indicates the positions where the presented B-scans were extracted. Green and orange ticks on the B-scans indicating extraction depths of the shallow and deep *en face*, respectively.

Figure 6 presents multi-scale three-dimensional OCT/OCM images of healthy right mandibular gingiva. Although the system’s high axial resolution provides the depth-resolved contrast necessary to reliably identify the macroscopic boundary between the epithelium and lamina propria in the cross-sectional OCT image (Figure 6(b.1)), the low lateral resolution of the 5× objective obscures fine structural details such as rete pegs shown in the *en face* images (Figure 6(b.2,b.3)). To resolve these delicate morphological features, Figure 6(c.1–d.3) display the reconstructed 10× and 20× OCT/OCM volumes, acquired using the aforementioned 3 × 3 and 6 × 6 mosaic protocol, respectively. These specific grid configurations (Table 2) were determined to ensure complete coverage of the 6 mm diameter tissue cassette window. The final reconstructed mosaics successfully yield wide-FOV areas of ~7 × 7 mm^2^ and ~7.2 × 7.2 mm^2^, respectively. By leveraging the custom-developed registration and 3D stitching algorithm, we ensure that the boundaries between sub-volumes are virtually imperceptible in both cross-sectional and *en face* images of the wide-FOV mosaic data.

The middle row (Figure 6(b.2,c.2,d.2)) presents *en face* images extracted at a depth of approximately 332 μm beneath the tissue surface (indicated by the green line in their corresponding cross-sectional images), corresponding to the rete peg layer. Rete pegs are epithelial extensions that project down into the underlying lamina propria. The distribution and shape of these rete pegs (indicated by the yellow asterisks) are clearly visualized; morphological alterations in these structures are important clinical indicators of pathology. Furthermore, we observed a strong correlation between architectural features in H&E-stained histology (Figure 6a) and the corresponding *en face* OCT/OCM images, particularly regarding the distribution of rete pegs.

The bottom row (Figure 6(b.3,c.3,d.3)) displays *en face* images at a deeper layer, approximately 570 μm below the tissue surface (indicated by the green line in their corresponding cross-sectional images), well into the lamina propria. In the high-resolution 20× *en face* image, the distal ends of the rete pegs projecting into the lamina propria can be distinctly identified as dark, dot-like structures (indicated by the yellow asterisks). Interestingly, an accidental needle puncture incurred during tissue handling (indicated by the yellow arrows across all panels) provided an unplanned but highly effective fiducial marker. This microscopic tissue defect is clearly identifiable in the H&E histology (Figure 6a) and can be precisely tracked through the 5×, 10×, and 20× *en face* OCT/OCM images. The system’s ability to correlate this unintended structural artifact across the varying OCT/OCM resolutions and corresponding H&E histology further validates the fidelity of the multi-scale imaging system.

However, for the 20× objective, the depth separation between the *en face* and the focal plane (~238 μm) exceeds its 120 μm DOF, the shallower plane (Figure 6(d.2)) resides out of focus. At this out-of-focus depth, the inherent optical distortion at the outer peripheral edges of the FOV of the high-NA objective becomes pronounced. Because our 3D mosaicking algorithm relies on spatial geometric consistency to perform multi-band blending, it cannot completely seamlessly merge these divergent, warped peripheral structures. Consequently, this physical optical limitation manifests as a visible stitching artifact (indicated by the white asterisks) in the peripheral quadrant of Figure 6(d.2).

**Figure 7:**
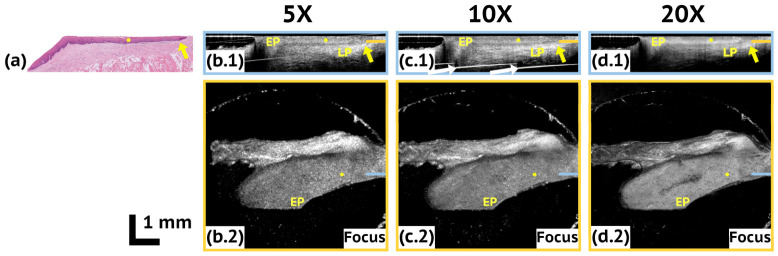
Multi-scale three-dimensional OCT/OCM images and corresponding H&E-stained histology of the healthy right lateral border of the tongue from a 67-year-old male. (**a**) Corresponding H&E-stained histology, which matches the cross-sectional images (**b.1**,**c.1**,**d.1**). (**b.1**,**c.1**,**d.1**) Cross-sectional OCT images acquired with the 5×, 10×, and 20× objectives, respectively. (**b.2**,**c.2**,**d.2**) *En face* OCT/OCM images extracted at a depth of 325 μm (near focal plane). Asterisks (*): normal rete pegs. Yellow arrows: indicate corresponding structural and pathological features successfully co-registered between the optical imaging and histology. White arrows: specular reflections. EP: epithelium; LP: lamina propria. Scale bars: 1 mm. The light-blue tick on the *en face* images indicates the positions where the presented B-scans were extracted. Orange ticks on the B-scans indicate the extraction depths of the *en face*.

Figure 7 presents multi-scale OCT/OCM images alongside the corresponding H&E histology images of healthy tongue tissue. While the low-resolution 5× cross-sectional image (Figure 7(b.1)) provides a macroscopic overview, it lacks the resolution necessary to clearly visualize distinct tissue layers. Consequently, high-resolution 10× and 20× volumetric scans were acquired using the aforementioned mosaicking protocols. In these high-resolution cross-sectional and *en face* reconstructions (Figure 7(c.1–d.2)), the boundary between the epithelium and the lamina propria is highly distinct and correlates well with the corresponding H&E histology (Figure 7a).

**Figure 8:**
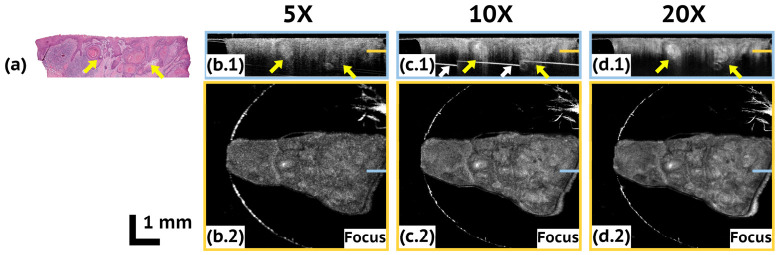
Multi-scale three-dimensional OCT/OCM images and corresponding H&E-stained histology of the SCC of the left buccal mucosa from a 48-year-old male. (**a**) Corresponding H&E-stained histology, which matches the cross-sectional OCT images (**b.1**,**c.1**,**d.1**). (**b.1**,**c.1**,**d.1**) Cross-sectional OCT images acquired with the 5×, 10×, and 20× objectives, respectively. (**b.2**,**c.2**,**d.2**) Corresponding *en face* OCT/OCM images extracted at a depth of 725 μm (near focal plane). Yellow arrows indicate corresponding structural and pathological features successfully co-registered between the optical imaging and histology. White arrows: specular reflections. Scale bars: 1 mm. The light-blue tick on the *en face* images indicates the positions where the presented B-scans were extracted. Orange ticks on the B-scans indicate the extraction depths of the *en face*.

**Figure 9:**
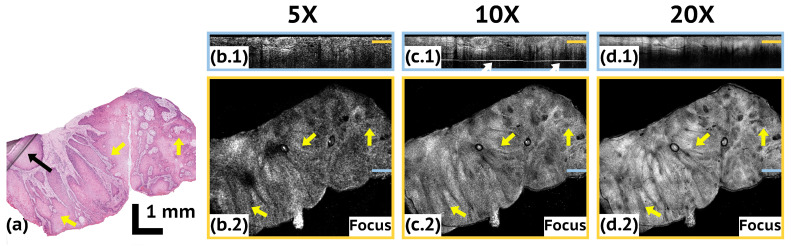
Multi-scale three-dimensional OCT/OCM images and corresponding H&E-stained histology of SCC of the left buccal mucosa from a 64-year-old male. (**a**) Corresponding H&E-stained histology, which matches the *en face* image (**b.2**,**c.2**,**d.2**). (**b.1**,**c.1**,**d.1**) Cross-sectional OCT images acquired with the 5×, 10×, and 20× objectives, respectively. (**b.2**,**c.2**,**d.2**) Corresponding *en face* OCT/OCM images extracted at a depth of 300 μm (near focal plane). Yellow arrows indicate corresponding structural and pathological features successfully co-registered between the optical imaging and histology. White arrows highlight specular reflections. The black arrow highlights the edge of the slide cover (**a**). Scale bars: 1 mm. The light-blue tick on the *en face* images indicates the positions where the presented B-scans were extracted. Orange ticks on the B-scans indicate the extraction depths of the *en face*.

Conversely, Figure 8 and Figure 9 illustrate multi-scale imaging of the SCC in buccal mucosa tissue from two different patients, acquired using the same mosaic scanning protocols. In contrast to the healthy tissue, the boundary between the epithelium and lamina propria is completely lost in the SCC OCT/OCM images. This optical feature directly reflects the underlying pathophysiology of SCC, where uncontrolled neoplastic proliferation and invasion disrupt the basement membrane, rendering the normal structural layers indistinguishable. Despite this architectural disruption, distinct morphological features, such as tumor nests and structural anomalies, can still be consistently identified and co-registered between the reconstructed high-resolution OCT/OCM images and the H&E-stained sections, as indicated by the yellow arrows.

## 4. Discussion

The development of an imaging modality capable of providing rapid, high-resolution, three-dimensional assessments of resected specimens represents a critical step toward advancing clinical management in surgical oncology. While long-wavelength OCT operating in the NIR-II window (1.68 μm) offers extended tissue penetration, its application to oral mucosa imaging has not previously been investigated. In this pilot study, we demonstrated a novel, fully automatic, multi-scale SS-OCT/OCM system to establish the technical foundation for rapid, wide-FOV imaging of oral mucosal specimens. Integrated with our custom-developed C++ imaging engine and user-friendly GUI [37], the system achieves automated, seamless resolution adjustment using objectives with three different magnifications. Following tissue cassette placement, a custom three-axis motorized assembly, comprising a two-axis motorized stage and a vertical-axis motorized stage, autonomously executes mosaic scanning, ensuring complete FOV coverage while dynamically compensating for the cover glass tilt to maintain continuous optical focus. Ultimately, the multi-scale, wide-FOV volumetric imaging presented in this study demonstrated its feasibility for rapid tissue imaging with a precise registration to corresponding histology images.

In practice, the volumetric stitching pipeline relies on a 10% FOV overlap to ensure sufficient structural features for cross-correlation. To optimize the registration efficiency, the initial spatial alignment is deterministically bounded by the optical FOV and the mechanical step size of the motorized stage. However, because specimen sizes inherently vary, the sub-volumes corresponding to the peripheral FOV often encompass feature-deficient regions due to, for example, sparse tissue, empty space, or artifacts such as air bubbles. In these regions, standard cross-correlation can fail, generating physically unrealistic or severely overestimated spatial shifts. To mitigate this issue, the pipeline evaluates the calculated translation against defined mechanical thresholds. If an estimated shift exceeds realistic bounds due to insufficient structural data, a fail-safe mechanism overrides the correlation peak and sets the translation to the motorized stage’s default displacement.

A primary challenge in correlating OCT images with the gold-standard H&E histology is the tissue shrinkage and morphological distortion that occur during chemical fixation and paraffin embedding. Previous studies have reported linear shrinkage rates of 10% to 20% in oral tissues [29]. Our custom-developed tissue cassette uses a proprietary design to preserve the structural integrity of the resected tissue by physically constraining it during both mosaic scanning and the subsequent fixation process (typically > 16 h). The cassette’s design incorporates a tilted cover glass to reject strong specular reflections from its surface, along with lateral perfusion ports that facilitate direct infiltration of 4% PFA. This design seamlessly bridges the optical scanning and histological preparation workflows. Because the tissue is fixed directly within the cassette post-scanning, this workflow guarantees a highly accurate, one-to-one spatial correlation between the optical volumes and the final H&E-stained sections.

The choice of a 1.68 μm swept-source laser was strategic to address the challenges inherent in OCT imaging for oral oncology management. Oral lesions, particularly oral SCC, are often characterized by hyperkeratotic surfaces that strongly scatter light. By operating within the NIR-II window, our system benefits from reduced Mie scattering relative to conventional 800 nm or 1.3 μm OCT systems, enabling clearer visualization of the basement membrane. However, the fundamental physics of the chosen wavelength presents a distinct trade-off. While the 1.68 μm NIR-II window effectively minimizes scattering, it also exhibits higher water absorption than traditional 1.3 μm OCT systems. Consequently, the overall achievable image depth is not drastically increased.

To contextualize the technological advancements of the platform developed in this study, Table 3 provides a summary of prior studies using OCT for ex vivo imaging of the oral mucosal specimens. Previous OCT systems have predominantly operated at 0.84 μm or 1.3 μm, using fixed-resolution optics and a constrained FOV. Unlike conventional single-scale setups, the developed platform integrates automated, multi-scale acquisitions (5×, 10×, and 20×) with advanced 3D stitching algorithms. This novel configuration overcomes traditional optical trade-offs, enabling seamless processing of multi-scale, high-resolution volumetric data across a large, flexible FOV. By providing comprehensive 3D datasets rather than fragmented 2D images, this integrated framework enables the systematic exploration of multi-scale pathological features using a 1.68 μm NIR-II wavelength-swept source—an approach previously unexplored in ex vivo mucosal specimens.

From a computational perspective, implementing the developed 3D volumetric stitching algorithm was essential to achieving true wide-FOV volumetric imaging. The algorithm accurately registers the acquired datasets while simultaneously correcting illumination heterogeneities caused by optical aberrations and changes in sample settings due to handling. However, the system’s overall performance is currently constrained by specific hardware limitations. First, while the gimbal-less dual-axis MEMS mirror provides a highly compact scanning solution, its open-loop operation inherently limits the maximum achievable scanning speed. Specifically, the high Q-factor of the MEMS mirror and the requisite low-pass filtering limit the linear bandwidth, creating a mechanical bottleneck during volumetric scanning. Consequently, an inherent trade-off exists between the A-scan rate of the light source and the mechanical sweeping frequency of the MEMS mirror. To accommodate this dynamic and ensure optimal image fidelity, the system intentionally employs a high scanning density along the fast axis.

A secondary hardware constraint involves the fundamental optical trade-offs of utilizing high-NA optics, particularly the 20× objective with its narrow DOF (~120 μm). To compensate for the cover-glass tilt during wide-FOV mosaicking, the vertical translation stage dynamically resets the sample elevation prior to each sub-volume acquisition. Consequently, while the focal plane tilts across individual sub-volumes, the maximum axial deviation at the peripheral edges is approximately 55 μm relative to the central focus plane. In addition, Figure 6(d.2) is technically outside the FWHM of the confocal gate (e.g., ~238 μm from the focal plane). However, as demonstrated by Lee et al. in 2013 [45], because the coherence gate effectively rejects out-of-focus scattered light, the OCM imaging at a distance from the FWHM of the confocal gate still retains structural fidelity that remains superior to the resolution of the 10× objective. While a default tilt is introduced to cover the glass of our custom tissue cassette to suppress the specular reflection, the variation in imaging resolution at the given depth of the surface-flattened imaging data is still present, which might affect the performance of the developed volumetric stitching algorithm. Future work will explore computational digital refocusing techniques [46] or modified optical hardware design to yield an extended depth of focus (EDOF) [47,48], thereby maintaining uniform lateral resolution and minimizing optical distortion across the entire volumetric dataset.

Notably, this temporal limitation is effectively mitigated by deploying the 10× objective, which yields sufficient structural clarity to identify the loss of the basement membrane boundary while requiring only <5 min to complete a full 3 × 3 mosaic scan. This accelerated acquisition provides a temporal advantage over standard intraoperative frozen section analysis, which typically requires 20 to 30 min, thereby demonstrating the potential for rapid intraoperative feedback.

Furthermore, translating this ex vivo platform into a routine intraoperative tool requires addressing several practical workflow challenges. First, the reproducibility of manual cassette loading is critical. Variations in how tissue is oriented or how much pressure is applied by the cover glass could introduce morphological artifacts. Developing a standardized loading protocol or a specialized clamping mechanism will be necessary for the operating staff. Second, real-world factors such as surface blood pooling heavily attenuate near-infrared light. While our current protocol includes a gentle rinsing step prior to loading, rigorous intraoperative fluid management is required to maintain high signal-to-noise ratios. Finally, integrating this system into clinical decision making presents a challenge as maxillofacial surgeons lack conventional training in interpreting OCT tomograms. To bridge this knowledge gap, our multiscale, co-registered datasets can be leveraged to develop a standardized imaging atlas.

To further accelerate acquisition and overcome these hardware constraints, future improvements could implement iterative-learning algorithms to optimize laser beam steering control [49]. More notably, deep learning (DL)-based optical super-resolution (SR) reconstruction technologies offer a powerful solution to bridge the gap between imaging resolution and acquisition time [50,51]. By using the 10× objective to rapidly acquire baseline intraoperative volumes, we can leverage a DL-SR model trained on paired low- and high-resolution images collected with the developed multi-scale OCT/OCM imaging system. Ultimately, this computational approach has the potential to simultaneously reconstruct the finer, 20×-equivalent structural details required by pathologists for final diagnostic confirmation, while reducing both data storage requirements and acquisition time by roughly 75%.

Ultimately, the primary objective of this wide-FOV, mobile imaging system is to establish a rigorous, one-to-one histopathological ground truth that minimizes tissue shrinkage artifacts. As shown in the results above, the current study demonstrated the feasibility of the developed multiscale imaging platform and also the volumetric stitching algorithm to provide rapid, volumetric, and multiscale imaging of the ex vivo oral mucosal specimens. As demonstrated in previous studies, the loss of the basement membrane could also be confirmed from the preliminary results of the 11 specimens (six normal oral mucosa and five SCC) imaged with the developed platform. It is important to note that this study serves as a proof-of-concept study and involves only imaging a small number of specimens with normal and SCC tissue pathologies. Future large-scale clinical studies will include more specimens with a broader spectrum of oral pathologies, for example, precancerous specimens with mild, moderate, and severe dysplasia. In addition, future studies will incorporate independent, blinded evaluations by board-certified oral pathologists to establish formal diagnostic criteria.

## 5. Conclusions

In this pilot study, we establish the feasibility of a novel, mobile, multi-scale, wide-FOV SS-OCT/OCM imaging platform for the rapid evaluation of ex vivo oral mucosal specimens. By using a 1.68 μm NIR-II source paired with multi-scale wide-FOV 3D stitching algorithms, our framework enables the systematic exploration of multi-scale pathological features—an approach previously unexplored in oral mucosa. The primary strength of this work lies in its robust hardware architecture and the in-house developed volumetric stitching pipeline. By physically constraining excised specimens within a custom tissue cassette, we mitigated macroscopic shrinkage artifacts. We achieved one-to-one spatial co-registration between our reconstructed optical volumes and the gold-standard H&E histopathology. Ultimately, this platform establishes a robust multi-scale imaging framework that will enable subsequent large-scale studies utilizing long-wavelength OCT for clinical oral oncology applications.

## Figures and Tables

**Table 1:** Performance of the different objectives employed in this study.

Objective Lens	5×	10×	20×
Depth of focus (DOF, mm)	1.780	0.456	0.120
Lateral resolution (FWHM, μm)	21.0	12.2	6.8
Field of view (FOV, mm^2^)	~6 × 6	~2.5 × 2.5	~1.3 × 1.3

**Table 2:** Mosaic scanning protocol settings employed in this study.

Objective Lens	10×	20×
Mosaic size (X × Y)	3 × 3	6 × 6
Mosaic FOV (mm^2^)	~7 × 7	~7.2 × 7.2

**Table 3:** Summary of previous works on OCT imaging of ex vivo oral mucosal specimens.

Study	Central Wavelength (μm)	Sweep Rate (kHz)	Lateral Resolution (μm)	Field of View (FOV, mm)	Scan Type	Assessment Metrics
Jerjes et al. [18] Hamdoon et al. [19]	1.310	20	<10	6	2D	Appearance
Adegun et al. [21,22]	1.305	20	8.4	6 × (5–10)	3D	Gradient
Hamdoon et al. [39]	1.310	20	<10	6	2D	Thickness
Sharma et al. [40]	0.840	10	30	—	2D	Thickness, Gradient
Jerjes et al. [41]	1.310	20	<10	6 × 6	3D	Thickness
Yang et al. [16]	1.310	100	17	6 × 6	3D	Appearance
Obade et al. [42]	1.325	16	25	10	2D	Appearance
Yang et al. [43]	1.310	100	17	6 × 6	3D	Classification
Zhou et al. [44]	0.840	—	15	0.5 × 1	3D	Mean gray value
Proposed system	1.688	90	21 (5×)/ 12.2 (10×)/ 6.8 (20×)	>7 × 7 *	Mosaic 3D	Appearance

* FOV is constrained by the dimensions of the tissue cassette cover glass. —: Not reported.

## Data Availability

Due to privacy and ethical restrictions, the datasets generated and presented during the current study are available from the corresponding author upon reasonable request.
